# Clinicopathological characteristics and *MYC* status determine treatment outcome in plasmablastic lymphoma: a multi-center study of 76 consecutive patients

**DOI:** 10.1038/s41408-020-0327-0

**Published:** 2020-05-29

**Authors:** Hanno M. Witte, Nadine Hertel, Hartmut Merz, Heinz-Wolfram Bernd, Veronica Bernard, Stephanie Stölting, Nikolas von Bubnoff, Alfred C. Feller, Niklas Gebauer

**Affiliations:** 1 Department of Hematology and Oncology, Federal Armed Hospital Ulm, Oberer Eselsberg 40, 89081 Ulm, Germany; 2 Department of Hematology and Oncology, University Hospital of Schleswig-Holstein, Campus Lübeck, Ratzeburger Allee 160, 23538 Lübeck, Germany; 3 Hämatopathologie Lübeck, Reference Centre for Lymph Node Pathology and Hematopathology, Lübeck, Germany

**Keywords:** B-cell lymphoma, B-cell lymphoma, Genetics research

Dear Editor,

Plasmablastic lymphoma (PBL) is a rare and aggressive B-cell disorder of post-germinal centre origin with features intermediate between high-grade B-cell lymphoma and plasma cell malignancies^1–3^. PBL recurrently arises in the elderly due to age-related immunosenescence, secondary to iatrogenic immunosuppresion, or in HIV-positive patients^4^. Due to the scarcity of the disease, patients are insufficiently represented in clinical trials, treatment is likely to be heterogeneous and translational research is challenging. Compared to other aggressive B-cell malignancies, PBL outcome is dismal and therapeutic options, especially in elderly and frail patients are highly limited, warranting the need for alternative therapeutic approaches^5^.

Beyond a proposed role of *PRDM1* mutations and recurrent, predominantly cytogenetic *MYC* alterations, favouring *MYC*-*IG* rearrangements, little is known about the biology and oncogenic drivers in PBL^6^. Previous clinicopathological studies emphasized the role of *MYC*-rearrangement status while a systematical assessment of clinical implications regarding *MYC* amplification has so far not been undertaken^7^. The antigen CD30, while pathogonomonic in anaplastic large cell lymphoma, is universally expressed is several types of lymphoma, including classical Hodgkin lymphoma (cHL), but more recent studies identified it as a potential therapeutic target in B-cell non-Hodgkin lymphoma subtypes as well^8^. Brentuximab vedotin as a potential targeted therapeutic approach is a monoclonal CD30 antibody-drug conjugate (monomethyl auritatine E), which is already approved, due to encouraging results for several indications, including relapsed and refractory (cHL) and CD30-positive peripheral T-cell lymphoma^9,10^.

In the current study, in order to advance our cognition of PBL biology and to broaden its potential therapeutic spectrum, we sought to assess clinicopathological baseline characteristics, *MYC* status, therapeutic variability and clinical outcome in the second largest PBL cohort published to date.

We retrospectively reviewed our institutional database to identify PBL patients whose biopsy specimen from initial diagnosis had been referred to the Reference center for Hematopathology University Hospital Schleswig Holstein Campus Lübeck and Hämatopathologie Lübeck for centralized histopathological panel evaluation between January 2000 and December 2018. Diagnosis was confirmed in a panel setting by three experienced hematopathologists (ACF, HM, and HWB) in accordance with the current edition of the WHO classification of tumors of the hematopoietic and lymphoid tissues^11^. Patients with insufficient follow-up or with insufficient or unrepresentative tissue samples were excluded. Antibodies and positivity cutoffs employed in the current study are summarized in Supplementary Table 1. Fluorescence in situ hybridization (FisH) for *MYC* was routinely performed, as described, wherever the biopsy (excision or needle-core) was of sufficient size and quality^12^.

In total, 76 consecutive patients with PBL (median age 63 years; range 26–91), were identified and assessed for clinicopathological and molecular baseline characteristics, therapy, and outcome. These characteristics of the study group are briefly summarized in Supplementary Table 2.

This present study was approved by the ethics committee of the University of Lübeck (reference-no 18–311) and conducted in accordance with the declaration of Helsinki. Patients had given written informed consent regarding routine diagnostic and academic assessment of their biopsy specimen at the Reference centre for Hematopathology and transfer of their clinical data.

Time to progression and overall survival (PFS, OS) were calculated from the date of initial diagnosis. Survival (PFS and OS) was initially estimated by means of the Kaplan–Meier method and univariate log-rank test. Characteristics with significant impact on either OS or PFS were subjected to a subsequent multivariate proportional hazard analysis. All statistical investigations were conducted using GraphPad PRISM 6 and SPSS 25 (IBM).

The median age of the study group was 63 years (26–91), 30 patients were HIV-positive while only two cases of PBL were found in post-transplant patients. The majority of patients presented with advanced stage (72.4% stage III/IV) disease and a clear male predominance was evaluable (77.6 vs. 22.4%). Of all patients, 53 (69,7%) were treated with CHOP-type therapy and 19 (25%) patients received none or less intensive protocols, including sole radiotherapy in palliative intent. Rituximab was administered in 19 patients although none of the cases were found to express CD20 by immunohistochemistry. Four elderly and frail patients with significant comorbidities (Charlson Comorbidity Index ≥ 7) refused any type of chemo- and/or radiotherapy and rapidly succumbed to progressive disease.

In this report, the overall response rate was 55.5% including 25% CR, which is in-line with previous reports, especially regarding the large proportion of elderly and frail patients in the current series^13^. A more refined delineation of the therapeutic regimens chosen in the current study supplemented with the corresponding clinical outcome data is provided in Supplementary Table 3 (full dosage regimens) and 4 (upfront dose-reductions, e.g., due to patient age or frailty).

Usage of novel agents in this study (e.g., proteasome inhibitors and imids) as part of salvage therapy regimens in a relapsed or refractory setting is briefly depicted in Supplementary Table 5.

There was no significant association between either *MYC* status or immunohistochemical positivity for CD30 and HIV-status (Supplementary fig. 1). This contradicts a recent report by Miao et al., who studied 13 PBL cases from china and supplemented their observations with a review of the literature^7^. Beyond the limited number of cases investigated in previous studies (*n* = 59), this circumstance may well be explained by the geographical heterogeneity regarding the composition of the study group (~40% of cases from asia), whereas we present an extensive central European multi-centre cohort (*n* = 63/76 with *MYC* data).

Beyond established prognostic factors such as R-IPI, age, ECOG performance status, and complete remission rate following initial therapy (CR-rate), we discovered *MYC* status (wild-type (wt) and amplification (amp) versus split (±amp)) to be a novel and significant prognosticator of OS with a trend towards a significant impact on PFS (*MYC*: split (±amp) vs. wt+amp; OS: *p* = 0.0318; HR: 2.071; CI: 1.082–3.967, multivariate *p* = 0.005; PFS: *p* = 0.0884; HR: 1.667; CI: 0.8902–3.120, multivariate *p* = 0.032) (Fig. 1). Conversely, immunohistochemical expression of CD30 as another potential prognosticator lost its impact on survival upon multivariate analysis (OS: *p* = 0.0155; HR: 0.5185; CI: 0.2885–0.9321, multivariate *p* = 0.285; PFS: *p* = 0.0617; HR: 0.6364; CI: 0.360–7.030, multivariate *p* = 0.350). Of note, our observations regarding the clinical impact of cytogenetic *MYC* alterations only reached statistical significance, upon combined analysis of *MYC* wild-type and *MYC* amplified cases versus *MYC* rearranged cases. This calculation appears legitimate, however, as *MYC* amplification alone did not influence clinical outcome in any way and *MYC* amplifications were further identified in both cases harbouring *MYC* rearrangements and unsuspicious *MYC* signals. In order to further characterize the clinical impact of our observations, we performed a cox-proportional hazard calculation, encompassing all prognostic factors, found to correlate with clinical outcome to a significant degree (*p* < 0.05) or, given the limited sample size and exploratory nature of the study, at least a trend towards the latter (*p* < 0.1) upon univariate analysis. Hereby, *MYC* split was found to independently predict inferior outcome in concert with IPI, stage, LDH and CR-rate, while predictive capabilities of CD30 expression were lost upon multivariate analysis. Both univariate as well as multivariate proportional hazard data regarding clinical outcome, correlated with clinicopathological characteristics are presented in Table 1.

**Fig. 1:**
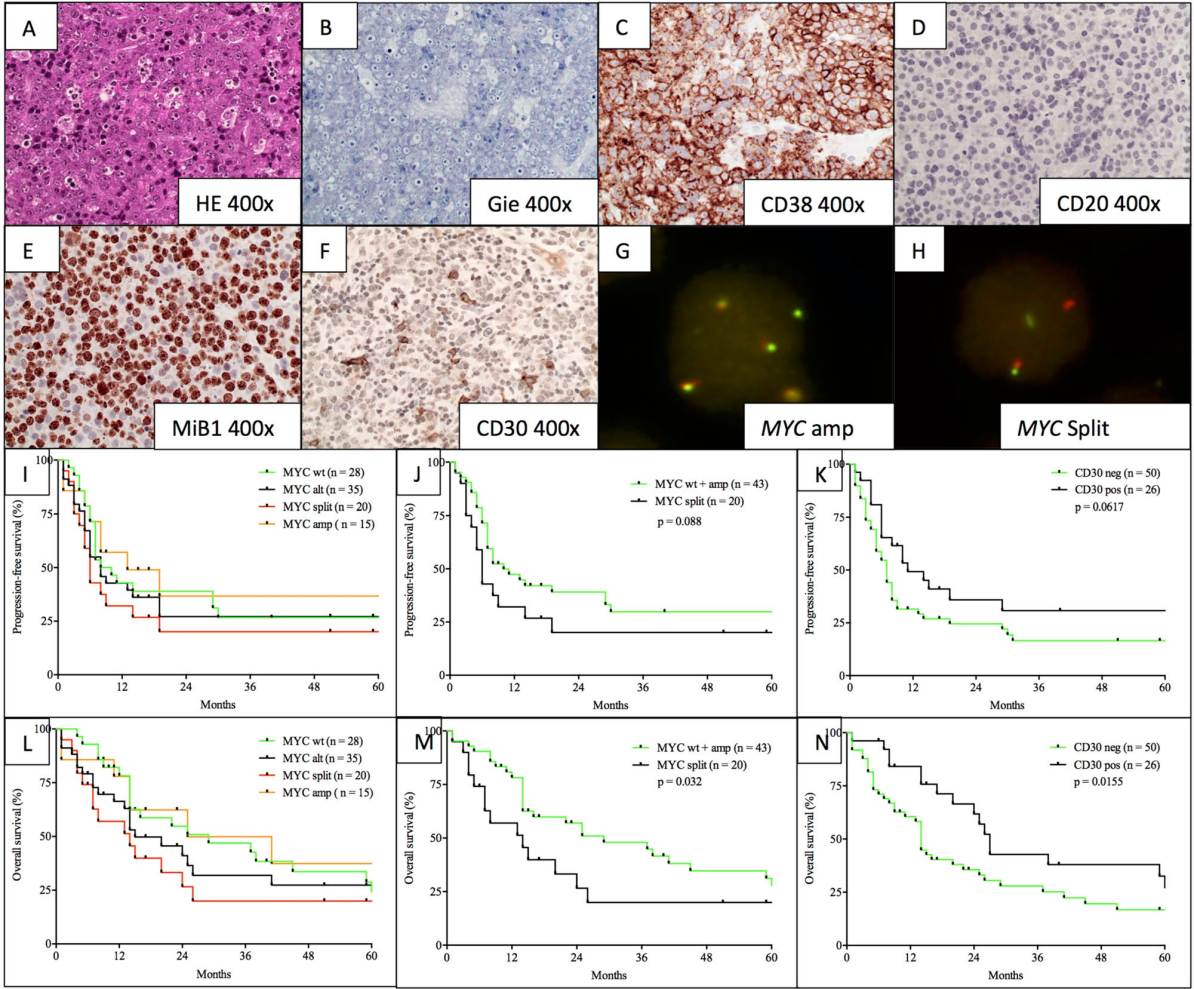
A representative case of plasmablastic lymphoma. Dense sheets of blast-like cells with elevated proliferative activity (HE, ×400; **a**; MiB1, ×400; **e**) and prominent plasmablastic/partially immunoblastic morphology (Giemsa, ×400; **b**). Immunophenotypically, the malignant cells are predominantly negative for most B-cell antigens like CD20 (×400, **d**), while several post-germinal and/or plasmacytic antigens (e.g., CD38) are strongly expressed (×400, **c**). As a potential therapeutic target as well as a novel means of prognostication, CD30 is expressed in a significant subset of PBL patients (CD30 ×400; ~15% positivity in the present case). By means of Fluorescence in situ hybridization for *MYC*, patients were subdivided into cases with wild-type *MYC*, *MYC* amplification (**g**) and *MYC* split ( ± concurrent amplification; **h**; in this case without concurrent amplification). Overview of clinical outcome according to cytogenetic *MYC* categories (**i** and **l**; wild-type (wt), split ( ± amp), amplification (amp) or any type of alteration (alt)) suggests a similar clinical course for patients harboring *MYC* amplifications when compared to *MYC* wild-type patients. Overall (**m** and **n**) and progression-free survival (**j** and **k**) according to *MYC* status (split vs. wt+amplification; OS: *p* = 0.0318; HR: 2.071; CI: 1.082–3.967; PFS: *p* = 0.0884; HR: 1.667; CI: 0.8902–3.120) and immunohistochemical expression of CD30 (OS: *p* = 0.0155; HR: 0.5185; CI: 0.2885–0.9321; PFS: *p* = 0.0617; HR: 0.6364; CI: 0.360–7.030) reveal significantly divergent clinical outcome (Gehan–Breslow–Wilcoxon test).

**Table 1:**
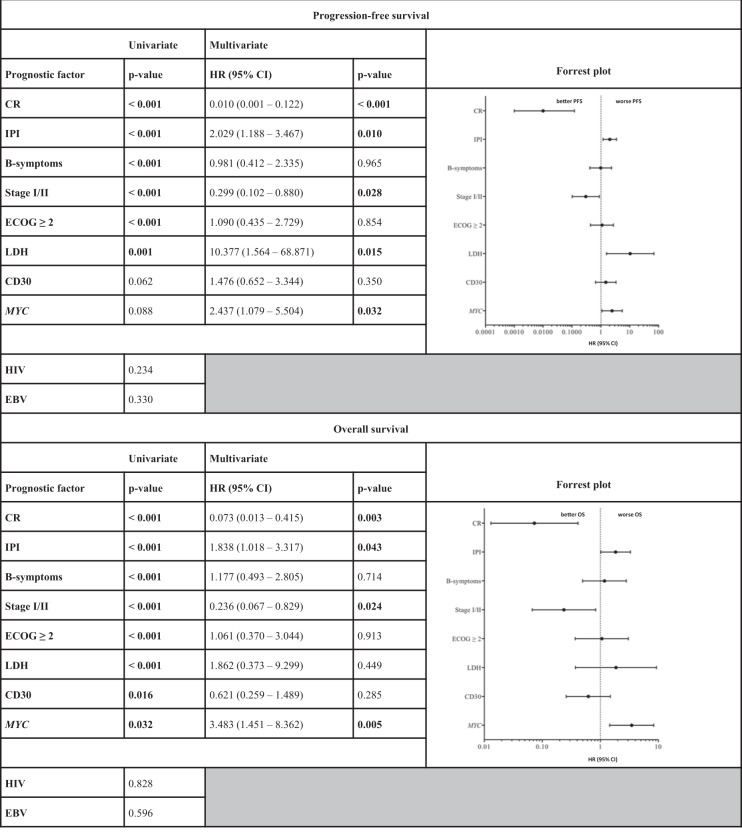
Univariate and sequential multivariate analysis of progression-free and overall survival, correlated with clinicopathological characteristics in PBL.

Despite intensive chemotherapy regimens and the introduction of novel agents (e.g., proteasome inhibitors and imids) on the basis of case studies, the prognosis of PBL has remained poor, with median overall survival ranging from 12–15 months^13,14^.

The essential observation to be drawn from this study, appears to be the prognostic capabilities of *MYC* split but not *MYC* amplification, which is well in keeping with recent findings in DLBCL, suggesting that *MYC* amplification alone does not predict an aggressive or adverse course of disease^15^. It therefore appears plausible to propose, that this concept applies to PBL, as well.

Of further note, we suggest that CD30, primarily based on its overall pronounced expression in PBL, may pose a potential therapeutic target in a seemingly already favourable subgroup of patients (Fig. 1). Given the broad range of proportion of positivity for CD30 (positivity cutoff 10%; range 0–70%) clinical data on treatment approaches, encompassing brentuximab vedotin, are of vital importance in the assessment of the significance of these observations. There appears to be room for cautious optimism, however, as findings from the ECHELON-2 study and others, suggest that CD30 positivity down to 10% is associated with significant susceptibility to brentuximab vedotin, superior to random vincristine treatment in combined immunochemotherapeutic approaches^10^. Besides the therapeutic targeting of CD30, future therapeutic concepts should also consider the consistently strong expression of CD38 and CD79b for which monoclonal antibodies and antibody-drug conjugates (e.g., daratumumab, polatuzumab vedotin) exist and were recently FDA approved for multiple myeloma and relapsed or refractory DLBCL.

Limitations of our study predominantly include its limited sample size and shortcomings inherent to the retrospective design of the study, such as the potential of a selection bias of indistinct extent especially when keeping in mind that clinical data were derived from routine medical records, which harbor the potential for fragmentary data. Apart from this, in a partially elderly and frail study group, the fraction of patients lost to follow-up due to non-lymphoma related death cannot be securely estimated from our data.

Despite these limitations, our analysis of the largest clinically and cytogenetically annotated cohort of PBL advances our insight into the clinical course of this rare yet aggressive disease and stresses the prognostic ramifications of specific *MYC* status while underlining the clinical implications of established prognosticators. In correlation with previously published data, we emphasize CD30 as a potential therapeutic target in a substantial subgroup of patients, which is therefore recommended to be further addressed in prospective trials.

## Supplementary information

Supplementary Table 1.

Supplementary Table 2.

Supplementary Table 3.

Supplementary Table 4.

Supplementary Table 5.

Supplementary Figure 1.

Reproducibility Checklist
