# Supplementary material for: Clinicopathological characteristics and MYC status determine treatment outcome in plasmablastic lymphoma: a multi-center study of 76 consecutive patients
Source: Blood Cancer J. 2020 May 29;10(5):63. doi: 10.1038/s41408-020-0327-0 (PMC7260224; doi:10.1038/s41408-020-0327-0)
Supplement: Supplementary file 1 — Supplementary Table 1. [file 41408_2020_327_MOESM1_ESM.docx]

**Supplementary Table 1.** Antibodies used

| **Antibody** | **Supplier** | **Clone** | **Positivity cutoff** |
| --- | --- | --- | --- |
| Bcl2  | Lab Vision | 100/D5 | 30% |
| Bcl6 | Dako | BG-B6p | 30% |
| CD10 | Menarini | 56C6 | 30% |
| CD20 | Dako | L26 | - |
| CD30 | Dako | BerH2 | 10%* |
| CD38 | Leica Biosystems | SPC32 | - |
| CD138 | Leica Biosystems | MI15 | - |
| CD56 | Leica Biosystems | CD564 | 10% |
| Kappa | Leica Biosystems | CH15 | - |
| Lambda | Leica Biosystems | SHL53 | - |
| MUM-1 (Irf4) | Dako | Mum 1P | 30% |
| Ki-67 | Dako | Mib-1 | - |
| * Employing and AUC-based algorithm as proposed by Budczies *et al.*, in order to determine the optimal cut-off values for the extent of CD30 positivity regarding survival, we were unable to detect a value of superior prognostic capabilities, beyond the established positivity of 10% of the tumor (data not shown). | | | |
