# Supplementary material for: Clinicopathological characteristics and MYC status determine treatment outcome in plasmablastic lymphoma: a multi-center study of 76 consecutive patients
Source: Blood Cancer J. 2020 May 29;10(5):63. doi: 10.1038/s41408-020-0327-0 (PMC7260224; doi:10.1038/s41408-020-0327-0)
Supplement: Supplementary file 2 — Supplementary Table 2. [file 41408_2020_327_MOESM2_ESM.docx]

| **Supplementary Table 2.** Baseline clinicopathological characteristics in PBL patients | |
| --- | --- |
| **Characteristics** | **Plasmablastic lymphoma (n = 76)** |
| **Age (yrs.; median + range)** | 63 (26-91) |
| **Sex** | |
| Female | 17 (22.4%) |
| Male | 59 (77.6%) |
| **HIV-status** | |
| HIV-positive | 30 (39.5%) |
| HIV-negative | 46 (60.5%) |
| **EBV-status** | |
| EBV-positive | 42 (55.3%) |
| EBV-negative | 34 (44.7%) |
| **HIV and EBV-positive** | 24 (31.6%) |
| **R-IPI** | |
| 0 | 7 (9.2%) |
| 1-2 | 18 (23.7%) |
| >2 | 51 (67.1%) |
| **Stage (Ann Arbor)** | |
| I | 8 (10.5%) |
| II | 13 (17.1%) |
| III | 16 (21.1%) |
| IV | 39 (51.3%) |
| **B-Symptoms** | |
| Yes | 45 (59.2%) |
| No | 31 (40.8%) |
| **Chromosomal aberrations** | |
| *MYC* overall (n = 63) | 35 (46.1%) |
| *MYC* amplification (n = 63) | 15 (19.7%) |
| *MYC* split(+/-amplification) (n = 63) | 20 (26.3%) |
| **CD30 by immunohistochemistry** | |
| Positive | 25 (32.9%) |
| Negative | 51 (67.1%) |
| **Extranodal sites** | |
| 0 | 11 (14.5%) |
| 1-2 | 60 (78.9%) |
| >2 | 5 (6.6%) |
| **ECOG PS** | |
| 0-2 | 55 (72.4%) |
| >2 | 21 (27.6%) |
| **LDH** | |
| Normal | 16 (21.1%) |
| Elevated | 60 (78.9%) |
| **CNS involvement at diagnosis** | |
| Yes | 0 (0.0%) |
| No | 76 (100.0%) |
| **Frontline therapy regimen** | |
| CHOP-like | 53 (69.7%) |
| R-based | 19 (25.0%) |
| Others | 19 (25.0%) |
| Refusal of treatment | 4 (5.3%) |
| **Frontline therapy response rates (n = 72)** | |
| CR | 18 (25.0%) |
| VGPR | 6 (8.3%) |
| PR | 16 (22.2%) |
| SD | 9 (12.5%) |
| PD | 23 (31.9%) |
| PBL, plasmablastic lymphoma; Yrs., years; CNS, central nervous system; LDH, Lactate dehydrogenase; ECOG; Eastern cooperative oncology group; GCB, germinal-center B-cell like; PS, performance status; CHOP, cyclophosphamide, doxorubicin, vincristine, prednisolone; R, rituximab; Others, other regimen (e.g. Bendamustine) or palliative cytoreductive treatment. | |
