# Supplementary material for: Clinicopathological characteristics and MYC status determine treatment outcome in plasmablastic lymphoma: a multi-center study of 76 consecutive patients
Source: Blood Cancer J. 2020 May 29;10(5):63. doi: 10.1038/s41408-020-0327-0 (PMC7260224; doi:10.1038/s41408-020-0327-0)
Supplement: Supplementary file 3 — Supplementary Table 3. [file 41408_2020_327_MOESM3_ESM.docx]

**Supplementary Table 3.** CHOP-like and R-based treatment regimens as frontline therapy.

| **Top 5 regimens used in the study.** | | | | | |
| --- | --- | --- | --- | --- | --- |
| **CHOP** | | | n = 28 | | |
| **R-CHOP** | | | n = 11 | | |
| **Bendamustin-based** | | | n = 8 (n = 3, in combination with R) | | |
| **CHOEP** | | | n = 8 | | |
| **R-CHOEP** | | | n = 3 | | |
|  | | | | | |
| **Characteristics** | **CHOP** | **R-CHOP** | **Bendamustin** | **CHOEP** | **R-CHOEP** |
| Male/female | 25 (89.3%)/  3 (10.7%) | 8 (72.7%)  3 (27.3%) | 4 (50.0%)  4 (50.0%) | 7 87.5%)  1 (12.5%) | 3 (100.0%)  - |
| Median age, years  (range) | 69.0  (29 – 80) | 48.0  (32 – 83) | 77.0  (70 – 86) | 44.5  (27 – 64) | 38.0  (26 – 55) |
| Response rates |  |  |  |  |  |
| CR | 5 (17.9%) | 4 (36.4%) | 1 (12.5%) | 5 (62.5%) | 2 (66.6%) |
| VGPR | 2 (7.1%) | 1 (9.1%) | - | 1 (12.5%) | - |
| PR | 9 (32.1%) | 3 (27.3%) | 1 (12.5%) | 1 (12.5%) | - |
| SD | 3 10.7%) | 2 (18.2%) | 3 (37.5%) | 1 (12.5%) | - |
| PD | 9 (32.1%) | 1 (9.1%) | 3 (37.5%) | - | 1 (33.3%) |
| CR120 | 4 (14.3%) | 3 (27.3%) | 1 (12.5%) | 5 (62.5%) | 2 (66.7%) |
| Cycles (median, range) | 6 (1 – 8) | 6 (2 – 8) | 4 (2 – 6) | 6 (4 – 6) | 4 (3 – 6) |
| Discontinuation | 13 (46.4%) | 2 18.2%) | 4 (50.0%) | 1 (12.5%) | 1 (33.3%) |
| Toxicity |  |  |  |  |  |
| Renal failure | 2 (7.1%) | 1 (9.1%) | - | 1 (12.5%) | - |
| Cardiotoxicity | 1 (3.6%) | - | 1 (12.5%) | 1 (12.5%) | - |
| Sepsis | 5 (17.9%) | 1 (9.1%) | 3 (37.5%) | 2 (25.0%) | - |
| Neutropenia | 9 (32.1%) | 4 (36.4%) | 4 (50.0%) | 3 (37.5%) | 1 (33.3%) |
| Polyneuropathy | 7 (25.0%) | 1 (9.1%) | - | 3 (37.5%) | 2 (66.7%) |
| CD 30 status |  |  |  |  |  |
| positive | 8 (28.6%) | 3 (27.3%) | - | 2 (25.0%) | 2 (66.7%) |
| weak | - | 4 (36.4%) | - | 1 (12.5%) | - |
| negative | 20 (71.4%) | 4 (36.4%) | 8 (100.0%) | 5 (62.5%) | 1 (33.3%) |
| HIV status |  |  |  |  |  |
| positive | 12 (42.9%) | 6 (54.5%) | - | 6 (75.0%) | 1 (33.3%) |
| negative | 16 (57.1%) | 5 (45.5%) | 8 (100.0%) | 2 (25.0%) | 2 (66.7%) |
| MYC status |  |  |  |  |  |
| negative | 10 (47.6%) | 4 (36.4%) | 4 (50.0%) | 3 (37.5%) | 2 (66.7%) |
| split | 4 (19.0%) | 5 (45.5%) | 1 (12.5%) | 3 (37.5%) | 1 (33.3%) |
| amplification | 7 (33.3%) | 2 (18.2%) | 1 (12.5%) | 1 (12.5%) | - |
| CHO(E)P, cyclophosphamide, daunorubicin, vincristine, (etoposide), prednisolone; CR, complete response; CR120, complete remission within 120 days; HIV, human immunodeficiency virus; PD, progressive disease; PR, partial remission; R, rituximab; SD, stable disease; VGPR, very good partial remission. | | | | | |
