# Supplementary material for: Clinicopathological characteristics and MYC status determine treatment outcome in plasmablastic lymphoma: a multi-center study of 76 consecutive patients
Source: Blood Cancer J. 2020 May 29;10(5):63. doi: 10.1038/s41408-020-0327-0 (PMC7260224; doi:10.1038/s41408-020-0327-0)
Supplement: Supplementary file 4 — Supplementary Table 4. [file 41408_2020_327_MOESM4_ESM.docx]

**Supplementary Table 4.** Upfront dose reduced CHOP-like treatment approaches used in the study.

| **ID** | **Regimen** | | **Cycles** | **Sex** | **Age** | **AA** | **HIV** | **BR** | **TTP** | **CD30** | ***MYC*** | **Last status** |
| --- | --- | --- | --- | --- | --- | --- | --- | --- | --- | --- | --- | --- |
| **01** | Mini-CHOP | | 4 | m | 86 | IVB | + | PD | 5 | - | - | Dfd |
| **Toxicity** | | Not applicable | | | | | | | | | | |
| **02** | R-Mini-CHOP | | 3 | m | 68 | IVB | - | PD | 4 | + | - | Dfd |
| **Toxicity** | | Not applicable | | | | | | | | | | |
| **03** | R-CHP | | 6 | m | 70 | IVB | - | CR | - | weak | - | sus. rem. |
| **Toxicity** | | Polyneuropathy, pneumonia, acute kidney injury with consecutive CRRT | | | | | | | | | | |
| AA, Ann-Arbor-Stage; BR, best response; CH(O)P, cyclophosphamide/ daunorubicin/(vincristine)/ prednisolone; CRRT, continuous renal replacement therapy; Dfd, died from disease; f, female; m, male; Mini, 50% dose reduction; PD, progressive disease; PR, partial remission; R, Rituximab; sus. rem., sustained remission; TTP, time to progress; +, positive; -, negative. | | | | | | | | | | | | |
