# Supplementary material for: Clinicopathological characteristics and MYC status determine treatment outcome in plasmablastic lymphoma: a multi-center study of 76 consecutive patients
Source: Blood Cancer J. 2020 May 29;10(5):63. doi: 10.1038/s41408-020-0327-0 (PMC7260224; doi:10.1038/s41408-020-0327-0)
Supplement: Supplementary file 5 — Supplementary Table 5. [file 41408_2020_327_MOESM5_ESM.docx]

**Supplementary Table 5.** Usage of novel agents in a relapsed or refractory setting focusing on the plasma cell myeloma-like aspect of PBL.

| **ID** | **Regimen** | | **Cycles** | **Sex** | **Age** | **AA** | **HIV** | **Prior lines** | **BR** | **TTP** | **CD30** | ***MYC*** | **Last status** |
| --- | --- | --- | --- | --- | --- | --- | --- | --- | --- | --- | --- | --- | --- |
| **01** | VAD | | 4 | f | 78 | IVB | - | - | PD | 5 | - | split | Dfd |
| **Toxicity** | | Antibody deficiency syndrome with consecutive pneumogenic sepsis | | | | | | | | | | | |
| **02** | R-Melphalan/Bortezomib | | 6 | f | 63 | IVA | - | - | PR | 29 | + | - | Dfd |
| **Toxicity** | | Severe pneumonia, requirement of invasive ventilation | | | | | | | | | | | |
| **03** | PAD+Melphalan+AutoSCT | | 3+1+1 | f | 66 | IVB | - | - | VGPR | 14 | - | split | Dfd |
| **Toxicity** | | Prolonged thrombocytopenia, mucositis | | | | | | | | | | | |
| **04** | VCD | | 2 | m | 77 | IVA | - | - | PD | 3 | - | split | Dfd |
| **Toxicity** | | Septic shock, multi organ failure | | | | | | | | | | | |
| **04** | Bortezomib | | 2 | m | 79 | IVB | - | 1 | PD | 2 | - | - | Dfd |
| **Toxicity** | | Pancytopenia | | | | | | | | | | | |
| **06** | CAD | | 4 | m | 87 | IVB | - | 1 | PR | 20 | + | split | Dfd |
| **Toxicity** | | Not applicable | | | | | | | | | | | |
| **07** | Pomalidomide+Dexamethasone | | 4 | m | 51 | IIA | + | 1 | PR | - | + | split | Sus. rem. |
| **Toxicity** | | Pancytopenia | | | | | | | | | | | |
| AA, Ann-Arbor-Stage; AutoSCT, autologous stem cell transplantation; BR, best response; CAD, cyclophosphamide/doxorubicin/dexamethasone, Dfd, died from disease; f, female; m, male; PAD, bortezomib/doxorubicin/dexamethasone; PD, progressive disease; PR, partial remission; R, Rituximab; sus. rem., sustained remission; TTP, time to progress; VAD, vincristine/doxorubicin/dexamethasone; VCD, bortezomib/cyclophosphamide/dexamethasone, +, positive; -, negative. | | | | | | | | | | | | | |
