# Supplementary figures and images for: Clinicopathological characteristics and MYC status determine treatment outcome in plasmablastic lymphoma: a multi-center study of 76 consecutive patients
Source: Blood Cancer J. 2020 May 29;10(5):63. doi: 10.1038/s41408-020-0327-0 (PMC7260224; doi:10.1038/s41408-020-0327-0)

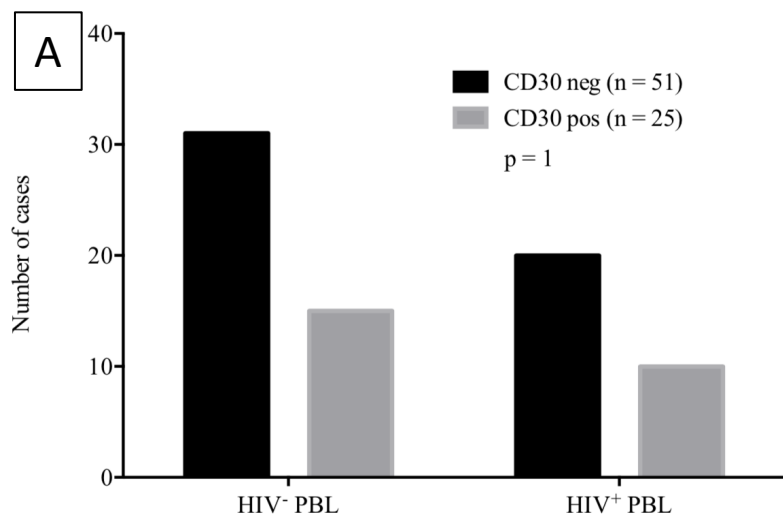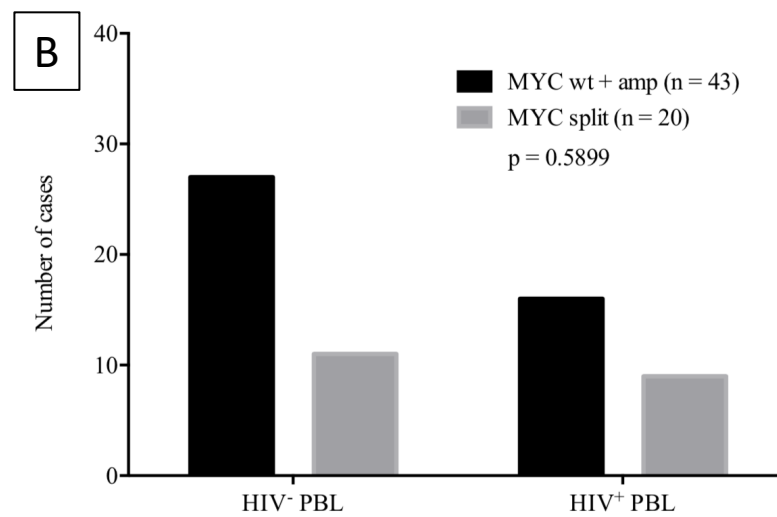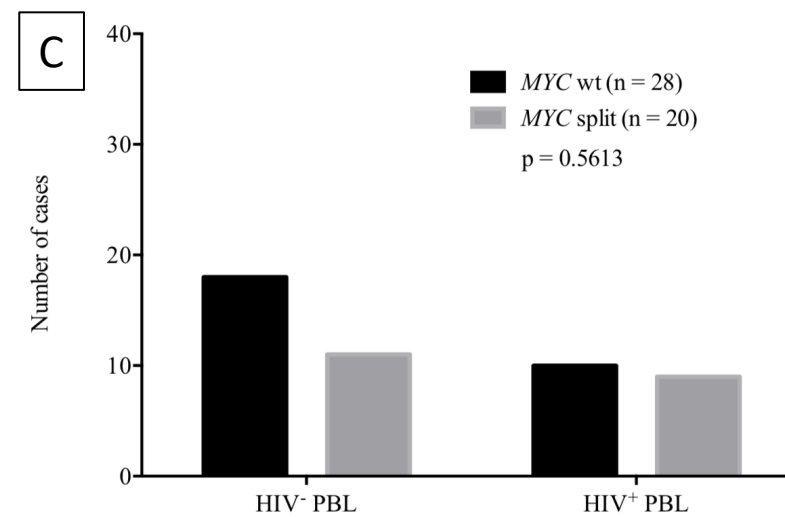

Supplement: Supplementary file 6 — Supplementary Figure 1. [file 41408_2020_327_MOESM6_ESM.pdf]
